# Psoriasis and medical ramifications: A comprehensive analysis based on observational meta-analyses

**DOI:** 10.3389/fmed.2022.998815

**Published:** 2022-08-29

**Authors:** Yun Zhou, Lixian Zhong, Lianli Shen, Sisi Chen, Qiuting Zeng, Leizhen Lai, Shaohui Tang

**Affiliations:** ^1^Department of Gastroenterology, The First Affiliated Hospital, Jinan University, Guangzhou, China; ^2^Department of Gastroenterology, The First Affiliated Hospital, Gannan Medical University, Ganzhou, China

**Keywords:** psoriasis, medical ramifications, meta-analysis, epidemiological evidence, quality

## Abstract

**Purpose:**

Based on a large number of systematic reviews and meta-analyses exploring the relationship between psoriasis and various health outcomes, we conducted an comprehensive analysis to assess the strength and evidence for the association between psoriasis and medical end-point ramifications in patients.

**Methods:**

We searched related meta-analyses, investigating the links between psoriasis and medical ramifications from three databases. All summary effect sizes, 95% CIs, heterogeneity, and small-study effects in the included meta-analyses were recalculated. We assessed the methodological quality of included articles with the AMSTAR 2 tool and graded the epidemiological evidence. Subgroup analysis based on the severity of psoriasis and study design were also performed.

**Results:**

A total of 38 articles comprising 85 unique meta-analyses were included in this study. Although 69 outcomes were statistically significant, only 8 outcomes (nonvascular dementia, ulcerative colitis, pediatric dyslipidemia, gestational diabetes, gestational hypertension, fracture, multiple sclerosis, and schizophrenia) showed a high quality of epidemiological evidence.

**Conclusion:**

We found that psoriasis increased the risk of 69 health outcomes, and 8 outcomes were graded as high-quality evidence. No evidence was found that psoriasis was beneficial for any medical end point. However, to verify our results, more large-sample, multi-center prospective cohort studies are needed.

## Introduction

Psoriasis, which can affect the skin, nails, and scalp, is a chronic, noncommunicable, painful disfiguring, and disabling disease, involving hyperproliferative keratinocytes and infiltration of T cells, dendritic cells, macrophages, and neutrophils (1, 2). So far, even with the proper treatments, psoriasis is still not cured but controlled (3). Affecting approximately 125 million adults and children all over the world, psoriasis results in serious global health issues and burdens (4). The overall prevalence of psoriasis ranges from 0.5 to 11.4% (5) and is higher in adults than in children. However, the majority of data on psoriasis prevalence are focused on a small number of countries (the U.K, the USA, and some European countries), causing that the prevalence of psoriasis is only known in 19% of countries worldwide (6). The psychosocial health of most patients with psoriasis may also receive a substantial and negative effect. Depression seems to be more common in patients with psoriasis (up to 20%) and even shows suicidal ideation extending to suicidal behavior when compared with the general population (7). The possibility of future development of psoriasis arthritis (PsA), another comorbidity of psoriasis, cannot be completely protected against the use of biological drugs (8). Therefore, psoriasis imposes a heavy burden on patients.

Until now, a large body of systematic reviews and meta-analyses investigated the association between psoriasis and certain medical ramifications. Except for psychiatric disorders, psoriasis seems to be linked with other comorbidities, including metabolic syndrome, uveitis, cardiovascular diseases (CVDs), and chronic kidney disease (9–12), which may further lead to impaired quality of life. However, most of the studies focused on a single health-related outcome, and to the best of our knowledge, no study has systematically summarized the strength of these relationships between psoriasis and multiple medical ramifications. A comprehensive analysis focuses on a specific topic and has the potential to provide the highest quality of evidence if performed and interpreted properly (13).

Consequently, we conducted this comprehensive analysis to gain a comprehensive overview of the existing published studies that investigated the association between psoriasis and health end point and assess its strength and validity. Our study shows that psoriasis has a major harmful effect on human health and can raise awareness of psoriasis.

## Methods

We have registered the protocol in PROSPERO (CRD42022306771). The study was conducted according to the preferred reporting items for systematic reviews and meta-analyses (PRISMA) regulations (14).

### Literature search

Using “psoriasis” AND “meta-analysis” OR “meta-analyses” OR “systematic review” as search terms, we searched PubMed, Web of Science, and Embase for relevant studies from the initiation to January 2022 with no other restrictions. We also screened the references in each selected article in order to avoid missing the potential meta-analyses.

### Study selection

Two authors selected independently the full texts for potentially eligible articles. Any discrepancies were discussed and resolved with ST. Studies were included if they met the following criteria:

(1) Meta-analysis was based on observational studies with quantitative analysis.
(2) Meta-analysis reported the association between psoriasis and the direct results on human health (risk of disease and mortality).

Studies were excluded if they met the following criteria:

(1) Meta-analysis only reported the relationship between psoriasis and indirect indicators (e.g., lipid levels and blood pressure).
(2) Studies were protocols, letters, and conference abstracts.
(3) Studies were only reviews.
(4) Psoriasis was not the exposure of interest.

When multiple meta-analyses investigated the same medical ramifications, we selected the one with the largest number of included primary studies.

### Data extraction

For each included meta-analysis, two authors (YZ and LZ) independently extracted the following information: name of the first author, publication year, health outcomes, severity of psoriasis, study design (case-control, cross-sectional, or cohort studies), number of studies included in each meta-analysis, number of participants and events, metric-type (OR, odds ratio; RR, relative risk; HR, hazard ratio), summary effect size, 95% confidence interval (CI) of the results, *P*-values for statistically significant level, Q-test, and Egger's test. All differences were discussed and resolved by consensus with a third person (ST).

### Data analysis

We used the extracted data obtained from the eligible studies to reanalyze each meta-analysis. The summary effect size and its 95% CI were all reestimated. We used the *I*^2^ metric and Cochran's Q-test to evaluate the heterogeneity. When the *I*^2^ is >50%, we choose the random effect model; otherwise, we choose the fixed effect model. Egger's regression test was used to evaluate the publication bias. A *P*-value of < 0.1 for heterogeneity and publication bias was considered statistically significant. If a meta-analysis reported the severity and type of study of psoriasis, we performed subgroup analyses on basis of these data.

### Evaluation of methodological quality and epidemiological evidence quality

We applied A Measurement Tool to Assess Systematic Reviews 2 (AMSTAR2), a robust and validated tool, to assess the methodological quality of each included study. AMSTAR2 contains 16 items, seven of which are key items, and it classifies the study quality into four ranks as follows: critically low, low, moderate, and high (15). To evaluate the evidence quality of the association between psoriasis and each medical end point, grading parameters that have been adapted in various studies (16–19) were used in this study. According to the criteria (precision of the estimate, number of cases, heterogeneity, and small-study effects), the strength of epidemiological evidence was categorized into high, moderate, weak, or not applicable. Two authors independently finished the above evaluations.

## Results

### Search result

**Figure 1:**
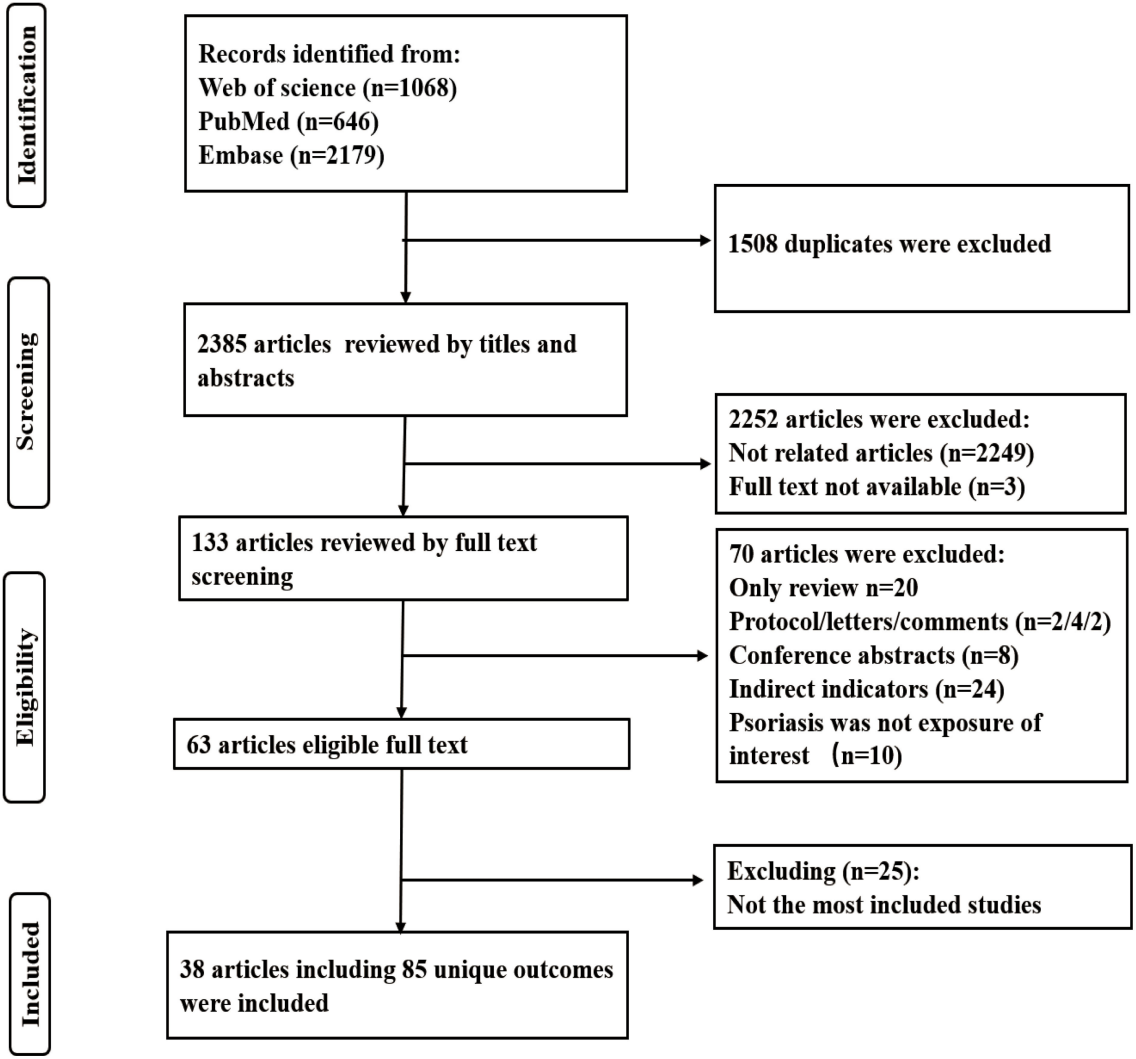
The PRISMA consort flow diagram of literature search and study selection.

**Figure 2:**
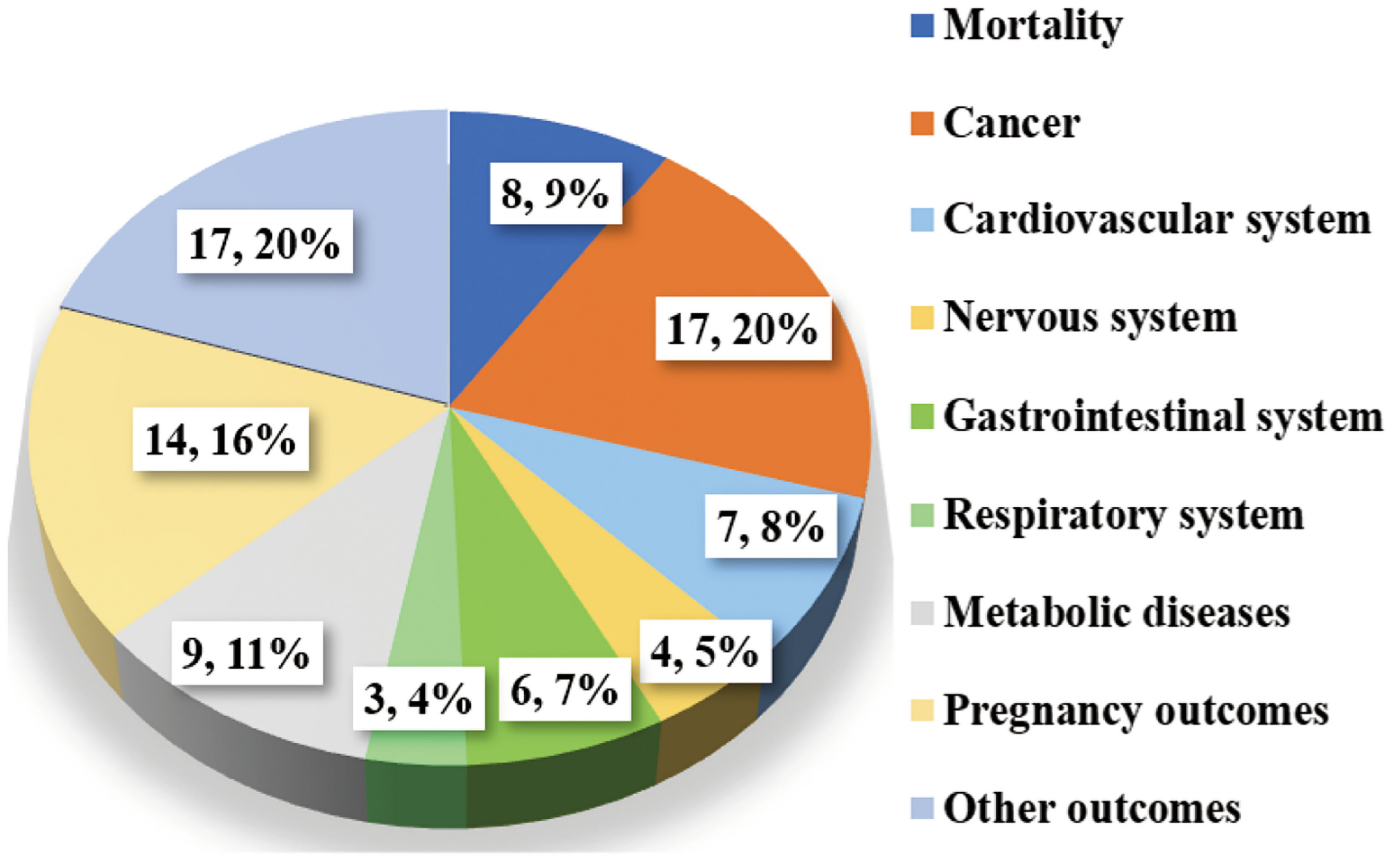
Map of medical end points associated with psoriasis.

Figure 1 shows the flowchart of the selection process. Overall, a total of 3,893 articles were identified from three databases (1,068 articles from Web of Science, 646 from PubMed, and 2,179 from Embase). After excluding 1,508 duplicates, we carefully screened for the inclusion and exclusion criteria and finally found 38 eligible articles with 85 unique medical end points in this study. The remaining articles were all published between 2012 and 2021. The characteristics of these meta-analyses are all shown in Supplementary Table 1. Medical end points related to psoriasis were involved in the following categories of diseases: mortality (*n* = 8), cancer (*n* = 17), cardiovascular system disease (*n* = 7), nervous system disease (*n* = 4), gastrointestinal system disease (*n* = 6), respiratory system disease (*n* = 3), metabolic disease (*n* = 9), pregnancy outcomes (*n* = 14), and other outcomes (*n* = 17) (Figure 2).

### Mortality

**Figure 3:**
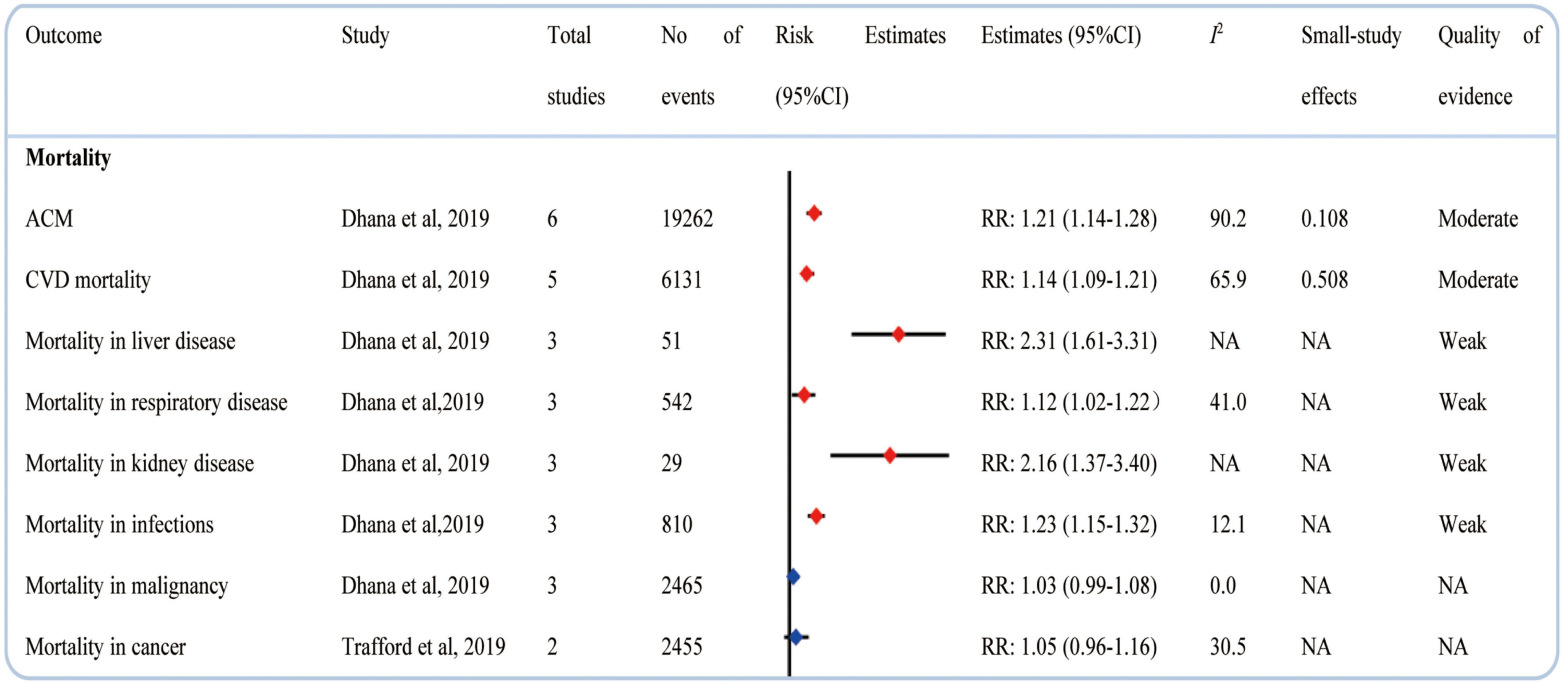
Associations between psoriasis and mortality.

A meta-analysis of 6 cohort studies showed that psoriasis and all-cause mortality (ACM) exhibited a positive correlation, with a RR of 1.21 (1.14–1.28) (20). Psoriasis was found to increase the mortality in CVD, liver disease, respiratory disease, kidney disease, and infections (20). But no significant associations between psoriasis and mortality in malignancy (20) and cancer (21) were found (Figure 3).

### Cancer

**Figure 4:**
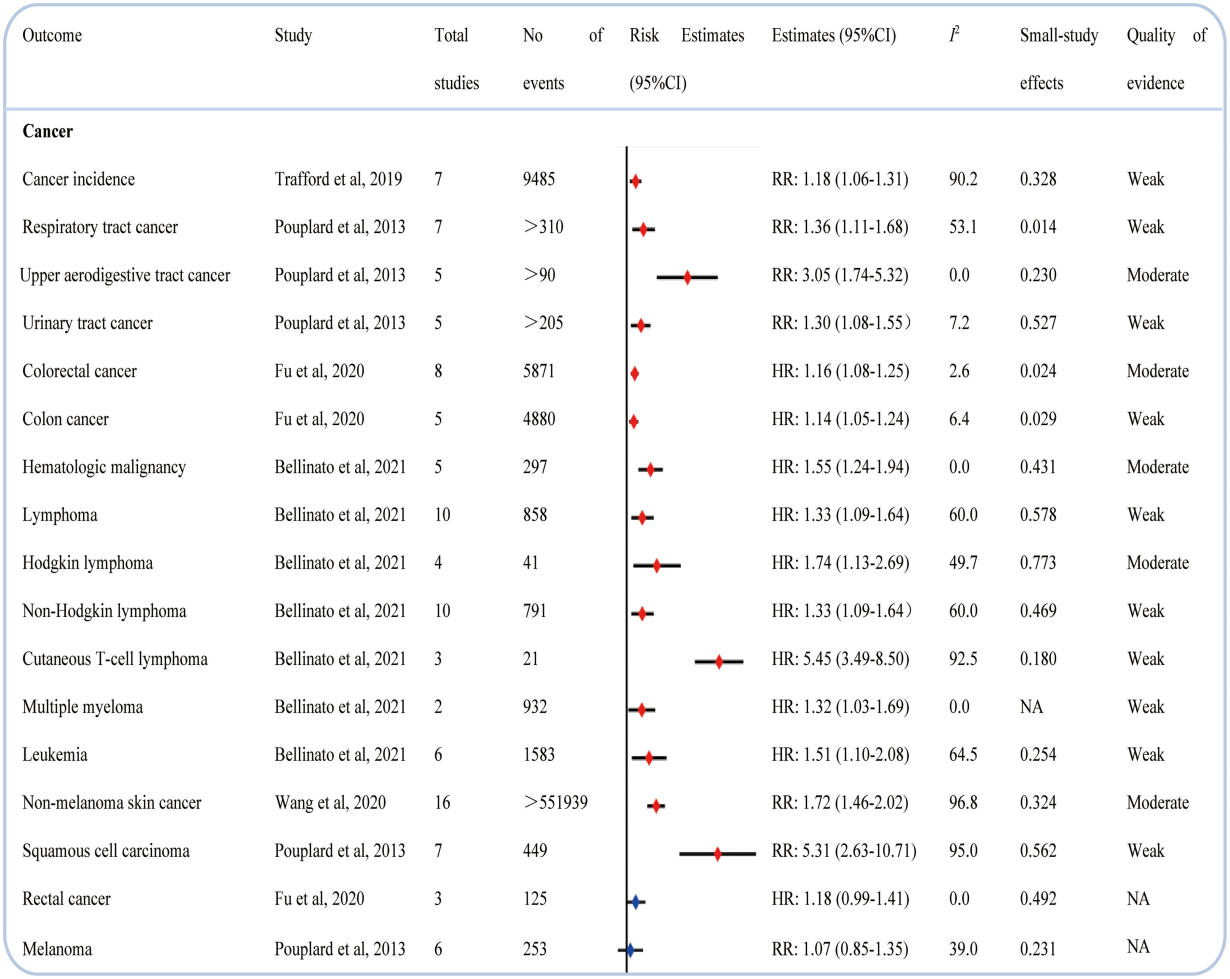
Associations between psoriasis and cancers.

Psoriasis increased the incidence of total cancer (21). Compared with people without psoriasis, those with psoriasis had an increased risk of respiratory tract cancer (22), upper aerodigestive tract cancer (22), urinary tract cancer (22), colorectal cancer (23), colon cancer (23), nonmelanoma skin cancer (24), and squamous cell carcinoma (22). Except for total hematological malignancy, we found that psoriasis was significantly correlated to evaluate the risk of lymphoma, Hodgkin lymphoma, non-Hodgkin lymphoma, cutaneous T-cell lymphoma, multiple myeloma, and leukemia (25). In addition, we found no relationship between psoriasis and rectal cancer (23) and melanoma (22) (Figure 4).

### Cardiovascular system

**Figure 5:**
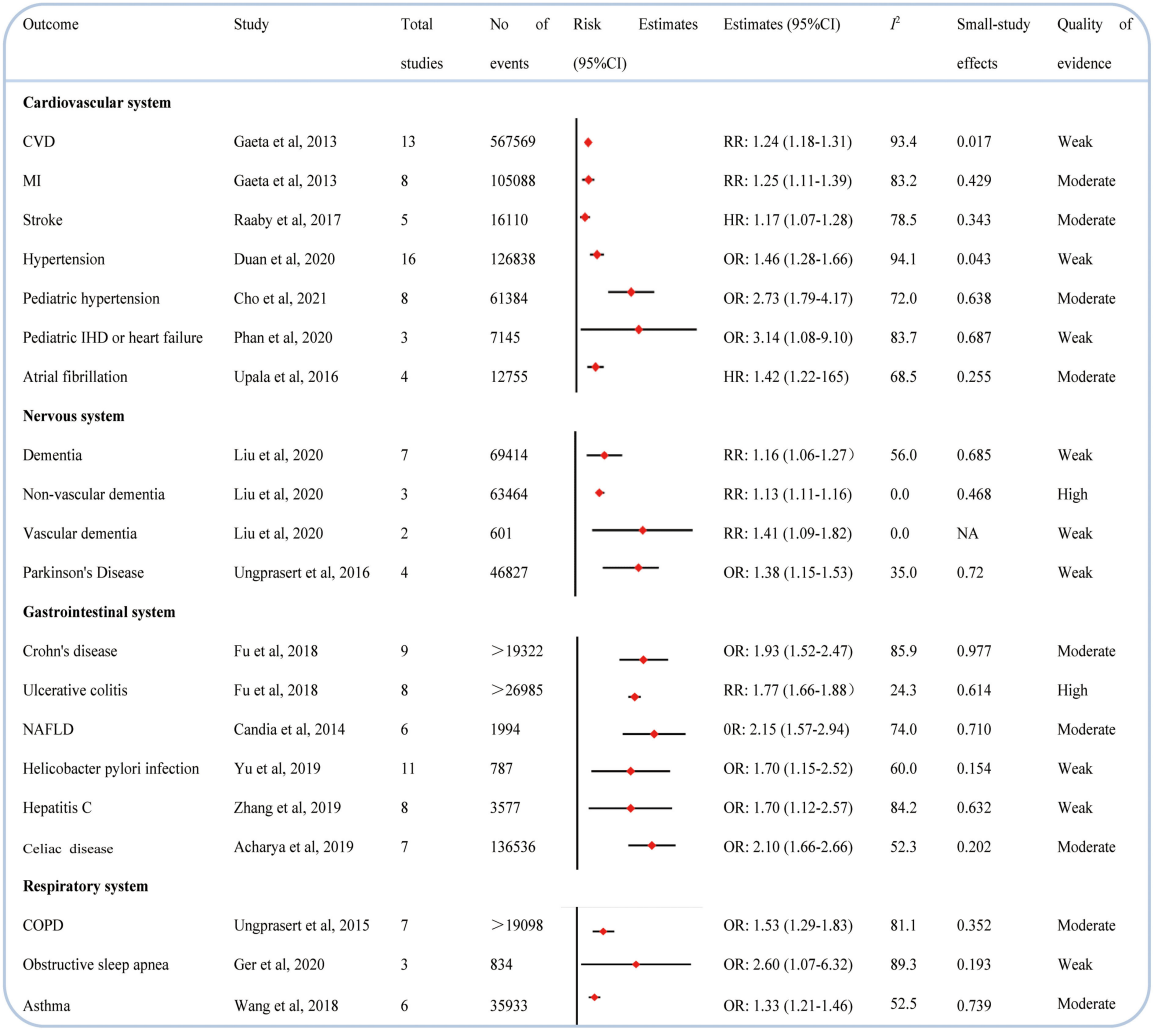
Associations between psoriasis and cardiovascular diseases (CVDs), nervous system, gastrointestinal system disease, and respiratory system disease.

In Figure 4, the adverse effects of psoriasis on cardiovascular system diseases are presented. We observed that 7 CVDs were linked to psoriasis. Previous data revealed that psoriasis evaluated the incidence of CVD (26), myocardial infarction (26), stroke (27), hypertension (28), pediatric hypertension (29), pediatric ischemic heart disease or heart failure (30), and atrial fibrillation (31) (Figure 5).

### Nervous system

Compared with individuals without psoriasis, those with psoriasis were more likely to develop a dementia (32), nonvascular dementia (32), vascular dementia, (32) and Parkinson's disease (33) in our study (Figure 5).

### Gastrointestinal system

Our data indicated that psoriasis can enhance the likelihood of developing Crohn's disease (34), ulcerative colitis (34), nonalcoholic fatty liver disease (NAFLD) (35), helicobacter pylori infection (36), hepatitis C (37), and celiac (38) (Figure 5).

### Respiratory system

As shown in Figure 5, only three medical ramifications (39–41) (i.e., chronic obstructive pulmonary disease, obstructive sleep apnea, and asthma) of the respiratory system were rated as psoriasis with an evaluated incidence.

### Metabolic diseases

**Figure 6:**
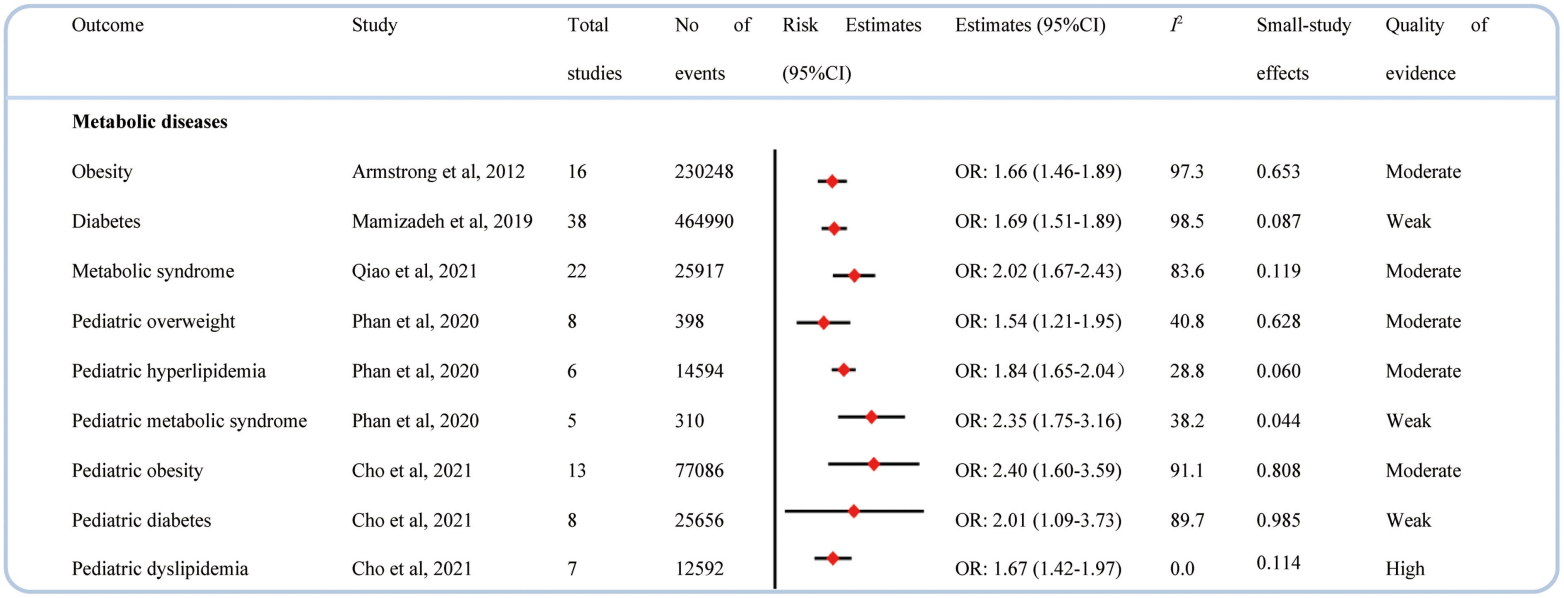
Associations between psoriasis and metabolic diseases.

Psoriasis had an adverse effect on metabolic disease in both adults and children. There were statistically significant associations found between psoriasis and incidence of obesity (42), diabetes (43), and metabolic syndrome (44) in adults. Sincerely, psoriasis also increased the risk of developing overweight (30), hyperlipidemia (30), metabolic syndrome (30), obesity (29), diabetes, (29) and dyslipidemia (29) in children (Figure 6).

### Pregnancy-related outcomes

**Figure 7:**
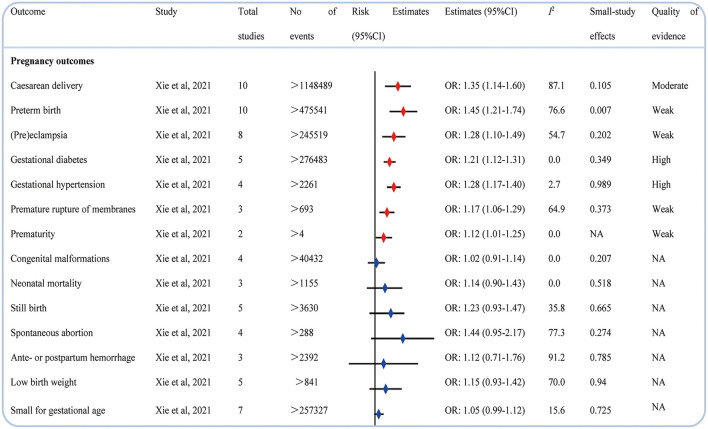
Associations between psoriasis and pregnancy outcomes.

A study assessed the possible association between psoriasis and pregnancy medical end points (45). Compared with pregnant women without psoriasis, those pregnant women with psoriasis seemed more likely to have harmful pregnancy-related outcomes such as cesarean delivery, preterm birth, (pre)eclampsia, gestational diabetes, gestational hypertension, premature of membranes, and prematurity; however, psoriasis was not associated with an increased risk of congenital malformations, neonatal mortality, stillbirth, spontaneous abortion, antepartum or postpartum hemorrhage, low birth weight, and small for gestational age (Figure 7).

### Other medical conditions

**Figure 8:**
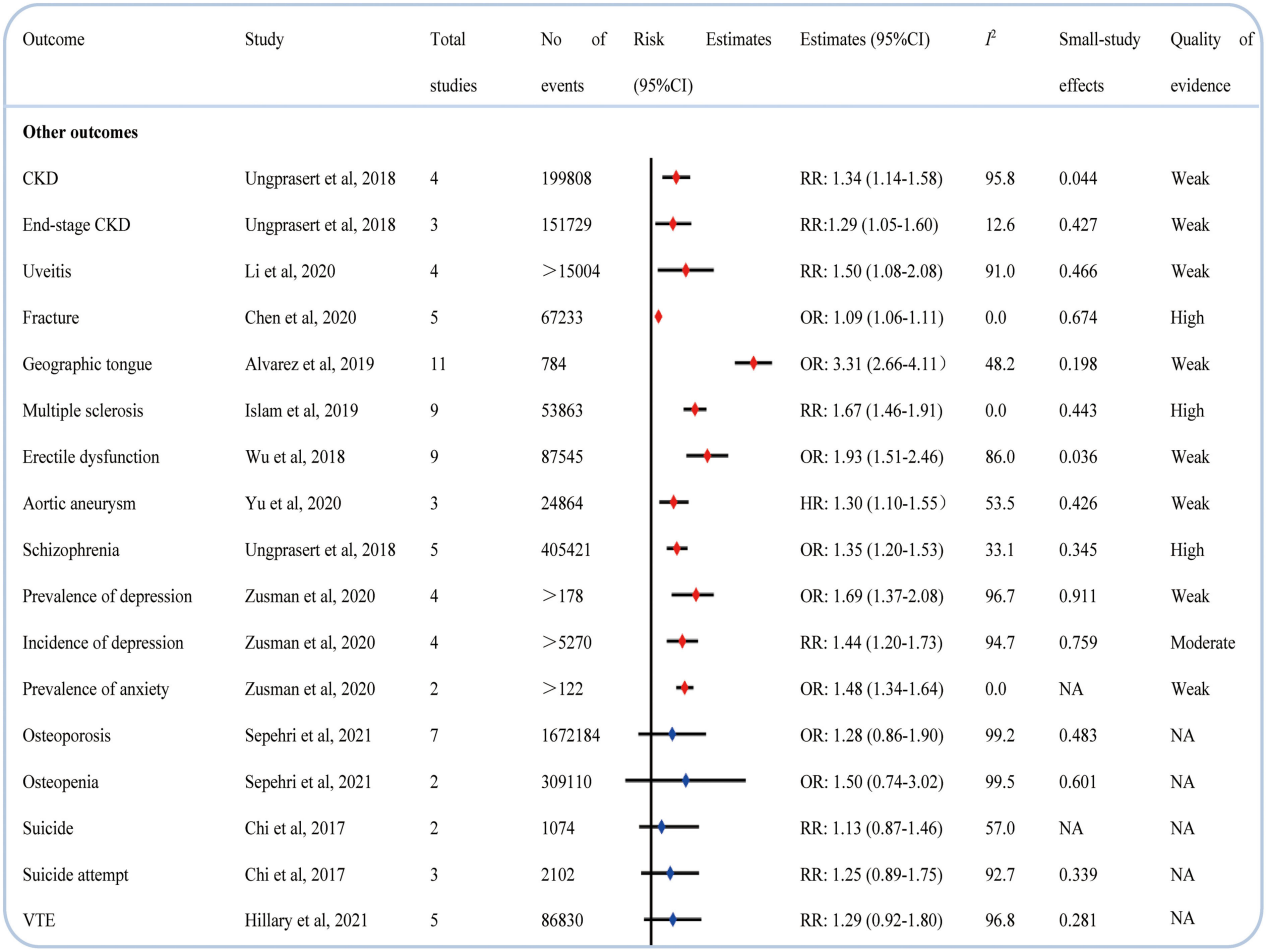
Associations between psoriasis and other outcomes.

We observed that psoriasis also evaluated the risk of CKD (46), end-stage CKD (46), uveitis (47), fracture (48), geographic tongue (49), multiple sclerosis (50), erectile dysfunction (51), aortic aneurysm (52), schizophrenia (53), prevalence and incidence of depression (54), and prevalence of anxiety (54). However, meta-analyses also reported the lack of significant correlation between psoriasis and osteoporosis (55), osteopenia (55), suicide (56), suicide attempt (56), and venous thromboembolism (VTE) (57) (Figure 8).

### Subgroup analysis

In a subgroup analysis of 22 meta-analyses that reported the severity of psoriasis, we found that only 6 outcomes were affected by the severity of psoriasis, while the remaining 16 outcomes were not (Table 1). We also conducted a subgroup analysis of 19 meta-analyses that concluded with cohort studies and case-control studies. The result showed that 16 medical end points were not influenced by the study design, whereas 3 medical end points were affected by the type of study (Table 2).

**Table 1:** Subgroup analysis results by severity of psoriasis.

**Outcome**	**References**	**Total studies**	**Estimates**	**Mild (studies)**	**Mild**	**Severe (studies)**	**Severe**
**Consistent effect**							
ACM	(20)	6	RR: 1.21 (1.14–1.28)	4	1.13 (1.09–1.16)	6	1.52 (1.35–1.71)
CVD mortality	(20)	5	RR: 1.14 (1.09–1.21)	3	1.05 (0.92–1.20)	4	1.38 (1.09–1.74)
Mortality in liver disease	(20)	3	RR: 2.31 (1.61–3.31)	1	2.00 (1.34–2.99)	3	3.97 (2.87–5.50)
Mortality in infections	(20)	3	RR: 1.23 (1.15–1.32)	1	1.41 (1.11–1.79)	3	1.58 (1.24–2.02)
Mortality in malignancy	(20)	3	RR: 1.03 (0.99–1.08)	1	1.02 (0.93–1.12)	3	1.21 (0.98–1.50)
Hodgkin lymphoma	(25)	4	HR: 1.74 (1.13–2.69)	1	1.42 (1.00–2.02)	1	3.18 (1.01–10.01)
Cutaneous T-cell lymphoma	(25)	3	HR: 5.45 (3.49–8.50)	2	3.70 (2.73–5.01)	2	9.67 (4.89–19.12)
Non-melanoma skin cancer	(24)	16	RR: 1.72 (1.46–2.02)	7	1.61 (1.25–2.09)	8	1.82 (1.38–2.41)
Stroke	(27)	5	HR: 1.17 (1.07–1.28)	5	1.10 (1.01–1.19)	5	1.38 (1.20–1.50)
Atrial fibrillation	(31)	4	HR: 1.42 (1.22–1.65)	2	1.22 (1.15–1.30)	2	1.51 (1.22–1.87)
Aortic aneurysm	(52)	3	HR: 1.30 (1.10–1.55)	3	1.23 (1.10–1.37)	3	1.55 (1.21–1,97)
Obstructive sleep apnea	(20)	3	OR: 2.60 (1.07–6.32)	1	1.36 (1.21–1.53)	1	1.53 (1.08–2.18)
Asthma	(41)	6	OR: 1.33 (1.21–1.46)	2	1.34 (1.14–1.57)	2	1.36 (1.03–1.80)
Obesity	(42)	16	OR: 1.66 (1.46–1.89)	4	1.46 (1.17–1.82)	5	2.23 (1.63–3.04)
Suicide	(56)	2	RR: 1.13 (0.87–1.46)	2	1.05 (0.84–1.31)	2	0.78 (0.45–1.35)
Suicide attempt	(56)	3	RR: 1.25 (0.89–1.75)	3	1.01 (0.51–2.01)	3	1.69 (1.00–2.84)
**Inconsistent effect**							
Mortality in kidney disease	(20)	3	RR: 2.16 (1.37–3.40)	1	2.20 (1.36–3.56)	3	2.28 (0.95–5.46)
Mortality in respiratory disease	(20)	3	RR: 1.12 (1.02–1.22)	1	1.36 (1.07–1.74)	3	1.06 (0.52–2.19)
Lymphoma	(25)	10	HR: 1.33 (1.09–1.64)	1	1.15 (0.97–1.36)	1	0.73 (0.28–1.90)
Non-Hodgkin lymphoma	(25)	10	HR: 1.33 (1.09–1.64)	1	1.15 (0.97–1.36)	1	0.73 (0.28–1.90)
Leukemia	(25)	6	HR: 1.51 (1.10–2.08)	1	0.95 (0.85–1.06)	1	0.88 (0.57–1.36)
Hypertension	(28)	16	OR: 1.46 (1.28–1.66)	4	1.09 (0.98–1.22)	5	1.13 (1.03–1.25)

ACM, all-cause mortality; CVD, cardiovascular disease.

**Table 2:** Subgroup analysis results by study design.

**Outcome**	**References**	**Total studies**	**Estimates**	**Cohort (studies)**	**Cohort**	**Case-control (studies)**	**Case-control**
**Consistent effect**							
CVD	(26)	13	RR: 1.24 (1.18–1.31)	4	1.18 (1.09–1.29)	4	1.24 (1.11–1.39)
MI	(26)	8	RR: 1.25 (1.11–1.39)	3	1.17 (0.99–1.38)	2	1.19 (1.05–1.36)
Hypertension	(28)	16	OR: 1.46 (1.28–1.66)	1	1.37 (1.29–1.45)	10	1.37 (1.14–1.64)
Dementia	(32)	7	RR: 1.16 (1.06–1.27)	5	1.13 (1.11–1.16)	1	1.25 (1.09–1.43)
Crohn's disease	(34)	9	OR: 1.93 (1.52–2.47)	4	2.53 (1.65–3.89)	5	1.70 (1.20–2.40)
Ulcerative colitis	(34)	8	OR: 1.77 (1.66–1.88)	4	1.72 (1.56–1.90)	4	1.75 (1.50–2.05)
Celiac	(38)	7	OR: 2.10 (1.66–2.66)	3	1.71 (1.48–1.98)	4	3.09 (1.92–4.97)
Asthma	(41)	6	OR: 1.33 (1.21–1.46)	3	1.34 (1.19–1.51)	3	1.26 (1.00–1.58)
Diabetes	(43)	38	OR: 1.69 (1.51–1.89)	12	1.40 (1.24–1.60)	15	1.89 (1.47–2.35)
Pediatric overweight	(30)	8	OR: 1.54 (1.21–1.95)	1	1.42 (0.44–4.53)	6	1.65 (1.05–2.60)
Pediatric hyperlipidemia	(30)	6	OR: 1.84 (1.65–2.04)	5	1.84 (1.65–2.04)	1	2.21 (0.33–14.64)
Cesarean delivery	(45)	10	OR: 1.35 (1.14–1.60)	8	1.28 (1.08–1.50)	2	2.22 (0.68–7.22)
Multiple sclerosis	(50)	9	RR: 1.67 (1.46–1.91)	5	1.55 (1.24–1.95)	4	2.01 (1.22–3.34)
Osteoporosis	(55)	7	OR: 1.28 (0.86–1.90)	2	1.04 (1.03–1.09)	3	1.38 (0.95–1.99)
Erectile dysfunction	(51)	9	OR: 1.93 (1.51–2.46)	1	1.27 (1.12–1.450	2	3.94 (2.87–5.41)
Schizophrenia	(46)	5	OR: 1.35 (1.20–1.53)	1	1.37 (1.01–1.86)	4	1.35 (1.19–1.54)
**Inconsistent effect**							
Parkinson's Disease	(33)	4	OR: 1.38 (1.15–1.53)	3	1.38 (1.20–1.58)	1	1.25 (0.51–3.06)
Hepatitis C	(37)	8	OR: 1.70 (1.12–2.57)	2	2.01 (1.67–2.42)	2	0.92 (0.14–6.00)
Helicobacter pylori Infection	(36)	11	OR: 1.70 (1.15–2.52)	3	1.64 (0.73–3.70)	4	1.20 (0.55–2.65)

CVD, cardiovascular disease; MI, myocardial infarction.

### Heterogeneity

Among 85 included meta-analyses, we could not reanalyze the heterogeneity for 2 meta-analyses due to the lack of detailed data from the original studies. A total of 33 meta-analyses showed low heterogeneity, while the remaining 50 showed high heterogeneity in our study.

### Strength of epidemiological evidence and AMSTAR2 results

**Figure 9:**
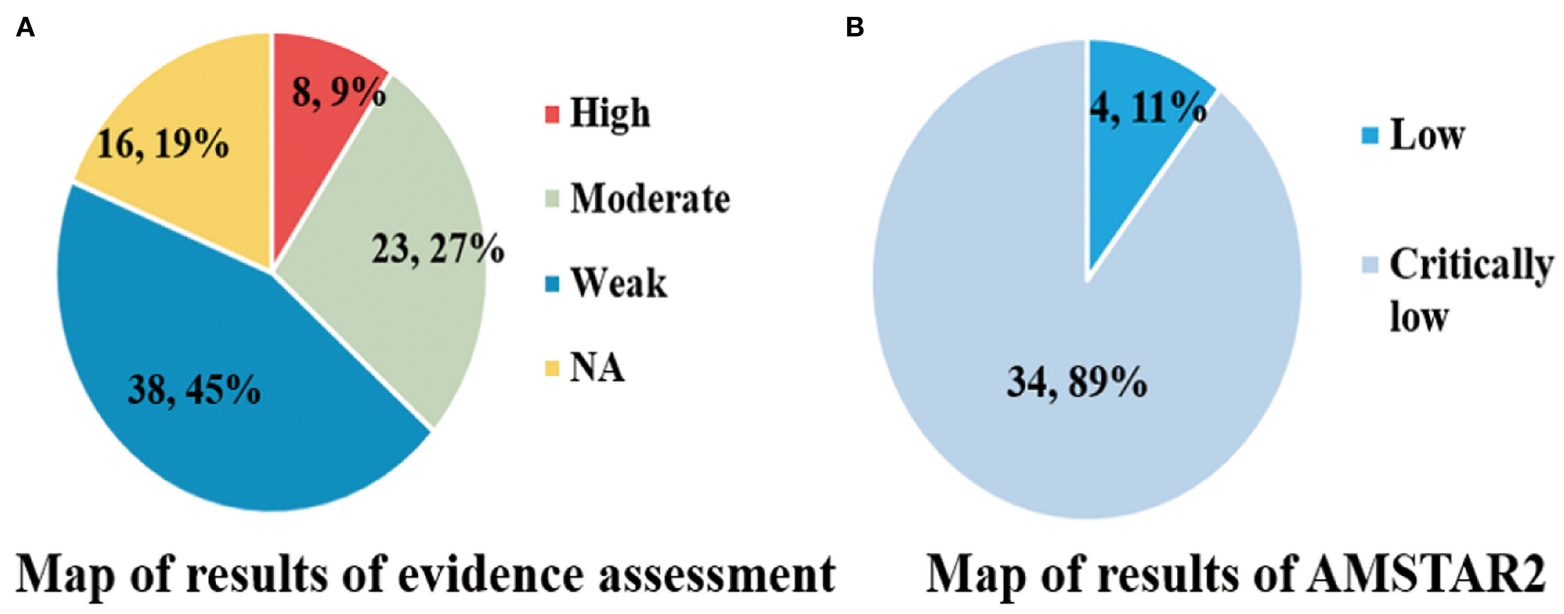
**(A,B)** Map of results of evidence assessment and AMSTAR 2.

According to the criteria (Supplementary Table 2), the assessment of epidemiological evidence was not applicable for 16 (19%) medical ramifications because the *P*-values for their summary effects were >0.05. Among 69 significant medical ramifications, 8 (9%), 23 (27%), and 38 (45%) medical ramifications were rated as high, moderate, and weak epidemiological evidence, respectively (Figure 9).

We observed that there was no high/moderate methodological quality according to the strict AMSTAR2 tool. Only 4 (11%) articles were rated as low, and the other 34 (89%) articles were rated as critically low (Figure 9). The detailed information of 16 items in AMSTAR2 and the results of specific quality scores are shown in Supplementary Table 3.

## Discussion

### Main findings and possible explanation

Based on the existing evidence from 38 articles that covered 85 unique medical end points, our study showed a broad overview of the association between psoriasis and other medical end points. The results covered mortality, cancer, cardiovascular system disease, nervous system disease, gastrointestinal system disease, respiratory system disease, metabolic disease, pregnancy outcomes, and other outcomes. We found that psoriasis had no beneficial effects on medical end points, while it was associated with an increased risk of 69 medical outcomes. However, the epidemiological evidence was graded as high only for 8 medical end points (i.e., nonvascular dementia, ulcerative colitis, pediatric dyslipidemia, gestational diabetes, gestational hypertension, fracture, multiple sclerosis, and schizophrenia). The remaining 61 associations were rated as moderate/weak in our study.

The management and treatment of psoriasis are complex, and there are currently data showing that Risankizumab, a biological agent, can improve psoriasis (58, 59). In particular, elderly patients represent an increasing proportion of patients with psoriasis. Biologics and small molecules seem to be a valuable option (60). In addition, anti-interleukin (IL)-23 therapies are assessed as safe and effective options for elderly patients (61). Therefore, early selection of appropriate drugs for psoriasis-related diseases is also very important.

Psoriasis is a complex disease involving many pathogeneses. The main pathogenesis of psoriasis highlighted the crosstalk between the innate and adaptive immune system, the central role of IL-23 and helper T cell type 17 responses, the role of TNFα and interferons, and the link to genetics (62). Our analysis found that psoriasis offers a harmful effect on nonvascular dementia, including Alzheimer's disease, with high epidemiological evidence. Another meta-analysis included eight observational studies that were consistent with our findings (63). Psoriasis and nonvascular dementia may share some genetic, immune, and inflammatory pathways. Ulcerative colitis, associated with psoriasis, also involves immune and inflammatory diseases and is of concern. In our study, the epidemiological evidence was graded as high for the association between them. There also exists common genetic abnormalities (chromosomal locus 6p21, IL23R, and IL12B) (64–66), immune dysfunction, and inflammation (IL17) (67, 68), or gut dysfunction (69) of disease progression both in both psoriasis and ulcerative colitis.

Dyslipidemia, hypertension, and diabetes in psoriasis are receiving more and more attention. We found that psoriasis increased the risk of pediatric dyslipidemia, gestational diabetes, and gestational hypertension with high-quality evidence. The correlation between cardiovascular risk factors (obesity, dyslipidemia, hypertension, and diabetes) and psoriasis has been arousing awareness and, consequently, with CVDs. In 2018, the American Heart Association and American College of Cardiology identified that chronic inflammatory diseases, such as psoriasis, act as a risk factor for CVD (70). It is not uncommon that psoriasis happens in children and adolescents as well as pregnant women. According to current screening guidelines, clinicians should screen for dyslipidemia from 9 years of age for psoriatic children and adolescents (71). Pregnant women with psoriasis had a higher risk to develop hypertension and diabetes than pregnant women without psoriasis in this study. For pregnant women, early treatment of psoriasis seems to be more important in order to avoid adverse pregnancy outcomes as well as CVD events. However, the underlying mechanism of the relationship between psoriasis and these cardiovascular risk factors remains unclear, and genetic susceptibility, cellular mediators, and the common inflammatory pathways seem to link these together.

Our study found a significant positive correlation between psoriasis and fracture with high epidemiological evidence, with a pooled RR of 1.09 (1.06–1.11). The following aspects might explain the mechanism. Long-term increased expression of inflammatory factors affects bone metabolism and exacerbates systematic bone loss (72). From the point of view of medicine, psoriatic patients taking methotrexate or ciclosporin can directly damage bone structure (73). Multiple sclerosis, which is considered to be an immune-mediated inflammatory disorder, has been reported as a comorbidity in psoriatic patients and vice versa (50, 74). With the high-quality evidence, psoriasis significantly increased the risk of multiple sclerosis in this study. These two diseases seem to have some biological similarities, involving common genes, immunity factors, and tumor necrosis factor, and these may mediate the inextricable link between them. Another notable correlation is between mental diseases and psoriasis. There has high epidemiological evidence that psoriasis enhanced the risk of schizophrenia in our study. Few possible interpretations can explain the result. T helper (Th17) is dysregulated in both diseases, which may share underlying etiology (75, 76). The proximity of chromosomes 6p21.3 and 6p22.2 to psoriasis and schizophrenia, respectively, may lead to the simultaneous transmission of these two susceptibility genes (77, 78). Both conditions are more likely to happen to the same person. Therefore, in the treatment of psoriasis, in addition, to pay attention to the treatment of the disease itself, it also needs to pay attention to these comorbidities.

In this study, the heterogeneity between studies has been observed in 50 meta-analyses. We think that the following confounding factors may be the causes of heterogeneity: age, severity of psoriasis, study design, geographical regions, and follow-up period. The methodological quality of studies included in this comprehensive analysis was all rated as low/critically low, and none was graded as high/moderate based on the AMSTAR2 criteria. We concluded that lack of protocol, list of excluded studies, founding source of primary studies, and the bias risk assessment were the main reasons for affecting the methodological quality. Only eight medical end points showed high-quality evidence. We observed that remarkable heterogeneity and small-study effects might contribute to the evidence rating downgrade in this study.

### Strength and limitations

To the best of our knowledge, this is the first time to systematically clarify the relationship between psoriasis and various medical end points. We applied robust search terms to identify eligible articles in three important databases to ensure the research result was as reliable as possible. Then, two authors independently screen the articles and extract the data from eligible studies. In the meanwhile, the AMSTAR2 tool and a strong evaluation method of evidence were used to assess the quality of the methodology of included studies and the epidemiological evidence. We suggest that this study can provide scientific evidence for arousing the awareness of psoriasis.

Inevitably, there are some limitations in our study. We included studies that focused on direct health-related outcomes, and thus we may have missed some data on indirect health-related results. We conducted the review based on the published studies with the largest number of included primary studies, and some individual studies might be missed, which could affect the results through selection bias. In the meanwhile, due to a lack of raw data, most of the meta-analyses failed to be conducted in subgroup analyses. Both prevalence and incidence of psoriasis are lower in children than in adults (79, 80). A systematic review conducted by Iskandar et al. suggests that there is a clear bimodal age pattern in psoriasis onset, showing the first and second peaks at around 30–39 and 60–69 years, respectively (81). Psoriasis occurs earlier in women than men, with a bimodal onset at the ages of 16–22 and 55–60 years (82). But the prevalence of psoriasis did not differ significantly between genders. Except for age, the prevalence of psoriasis is unequally distributed across a geographical area. According to some studies, the prevalence of psoriasis is unequally distributed across geographical regions, with the highest prevalence in high-income countries and prevalence ranging from 0.1% in east Asia to 1.5% in western Europe (6). Unfortunately, many of the included meta-analyses lacked data on these two aspects, and there was no way to perform subgroup analyses across age and geographic regions to provide more reliable and accurate results in our study.

## Conclusion

We found high-quality evidence showing that psoriasis is adverse for nonvascular dementia, ulcerative colitis, pediatric dyslipidemia, gestational diabetes, gestational hypertension, fracture, multiple sclerosis, and schizophrenia. No evidence was found to be beneficial for medical end points in our comprehensive analysis. Nonetheless, more large-sample, multi-center prospective cohort studies are needed to verify our results.

## Data availability statement

The original contributions presented in the study are included in the article/Supplementary material, further inquiries can be directed to the corresponding author.

## Author contributions

YZ, LZ, LS, and ST were involved in the conception and design of the study and drafted the manuscript. YZ, LZ, LS, SC, QZ, and LL contributed to the acquisition and analysis of the data. YZ and LZ interpreted the results. All authors read and approved the final manuscript.

## Conflict of interest

The authors declare that the research was conducted in the absence of any commercial or financial relationships that could be construed as a potential conflict of interest.

## Publisher's note

All claims expressed in this article are solely those of the authors and do not necessarily represent those of their affiliated organizations, or those of the publisher, the editors and the reviewers. Any product that may be evaluated in this article, or claim that may be made by its manufacturer, is not guaranteed or endorsed by the publisher.

## Abbreviations

PsA: psoriasis arthritis
CVD: cardiovascular disease
ACM: all-cause mortality
RR: relative risk
NAFLD: nonalcoholic fatty liver disease
CKD: chronic kidney disease
VTE: venous thromboembolism.
