# Supplementary material for: Psoriasis and medical ramifications: A comprehensive analysis based on observational meta-analyses
Source: Front Med (Lausanne). 2022 Aug 29;9:998815. doi: 10.3389/fmed.2022.998815 (PMC9465012; doi:10.3389/fmed.2022.998815)
Supplement: Supplementary file 1 [file Table_1.pdf]

Supplementary Table 1. Characteristics of the unique meta-analyses investigating the associations between psoriasis and multiple health outcomes.

Health outcomes	Author, year	Case-control/cross-sectional/cohort/total studies (n)	Participants (n)	Events (n)	Type of metric	Effect size		Heterogeneity		Small-study effect
						95% CI	*P* Value	I^2	*P* Value	
**Mortality**										
ACM	Dhana et al, 2019	0/0/6/6	299374+general population	19262	RR	1.21 (1.14-1.28)	0.000	90.2	＜0.001	0.108
CVD mortality	Dhana et al, 2019	0/0/5/5	162469+general population	6131	RR	1.14 (1.09-1.21)	0.000	65.9	0.020	0.508
Mortality in liver disease	Dhana et al, 2019	0/0/3/3	39074+general population	51	RR	2.31 (1.61-3.31)	0.000	NA	NA	NA
Mortality in respiratory disease	Dhana et al, 2019	0/0/3/3	119241+general population	542	RR	1.12 (1.02-1.22）	0.015	41.0	0.193	NA
Mortality in kidney disease	Dhana et al, 2019	0/0/3/3	1376+general population	29	RR	2.16 (1.37-3.40)	0.000	NA	NA	NA
Mortality in infections	Dhana et al, 2019	0/0/3/3	119241+general population	810	RR	1.23 (1.15-1.32)	0.000	12.1	0.286	NA
Mortality in malignancy	Dhana et al, 2019	0/0/3/3	119241+general population	2465	RR	1.03 (0.99-1.08)	0.111	0.0	0.701	NA
Mortality in cancer	Trafford et al, 2019	0/0/2/2	119241	2455	RR	1.05 (0.96-1.16)	0.075	30.5	0.230	NA
**Cancer**										
Cancer incidence	Trafford et al, 2019	0/0/7/7	259075	9485	RR	1.18 (1.06-1.31)	0.024	90.2	0.000	0.328
Respiratory tract cancer	Pouplard et al, 2013	0/0/7/7	27815	＞310	RR	1.36 (1.11-1.68)	0.003	53.1	0.047	0.014
Upper aerodigestive tract	Pouplard et al, 2013	0/0/5/5	24770	＞90	RR	3.05 (1.74-5.32)	0.000	0.0	0.993	0.230
Urinary tract cancer	Pouplard et al, 2013	0/0/5/5	24898	＞205	RR	1.30 (1.08-1.55）	0.005	7.2	0.366	0.527
Colorectal cancer	Fu et al, 2020	0/0/8/8	＞1406691	5871	HR	1.16 (1.08-1.25)	0.000	2.6	0.410	0.024
Colon cancer	Fu et al, 2020	0/0/5/5	1201262	4880	HR	1.14 (1.05-1.24)	0.010	6.4	0.371	0.029
Hematologic malignancy	Bellinato et al, 2021	0/0/5/5	296001	297	HR	1.55 (1.24-1.94)	0.000	0.0	0.676	0.431
Lymphoma	Bellinato et al, 2021	0/0/10/10	＞979519	858	HR	1.33 (1.09-1.64)	0.006	60.0	0.008	0.578
Hodgkin lymphoma	Bellinato et al, 2021	0/0/4/4	＞888531	41	HR	1.74 (1.13-2.69)	0.000	49.7	0.113	0.773
Non-Hodgkin lymphoma	Bellinato et al, 2021	0/0/10/10	＞979719	791	HR	1.33 (1.09-1.64）	0.006	60.0	0.008	0.469
Cutaneous T-cell lymphoma	Bellinato et al, 2021	0/0/3/3	＞16678	21	HR	5.45 (3.49-8.50)	0.000	92.5	0.000	0.180
Multiple myeloma	Bellinato et al, 2021	0/0/2/2	901802	932	HR	1.32 (1.03-1.69)	0.030	0.0	0.820	NA
Leukemia	Bellinato et al, 2021	0/0/6/6	999281	1583	HR	1.51 (1.10-2.08)	0.012	64.5	0.015	0.254

Supplementary Table 1. (continued)

Health outcomes	Author, year	Case-control/cross-sectional/cohort/Total studies (n)	Participants (n)	Events (n)	Type of metric	Effect size		Heterogeneity		Small-study effect
						95% CI	*P* Value	I^2	*P* Value	
Non-melanoma skin cancer	Wang et al, 2020	0/0/16/16	16059500	＞551939	RR	1.72 (1.46-2.02)	0.000	96.8	0.000	0.324
Squamous cell carcinoma	Pouplard et al, 2013	0/0/7/7	27150	449	RR	5.31 (2.63-10.71)	＜0.0001	95.0	＜0.0001	0.562
Rectal cancer	Fu et al, 2020	0/0/3/3	32270	125	HR	1.18 (0.99-1.41)	0.062	0.0	0.425	0.492
Melanoma	Pouplard et al, 2013	0/0/6/6	26150	253	RR	1.07 (0.85-1.35)	0.580	39.0	0.150	0.231
**Cardiovascular system**										
CVD	Gaeta et al, 2013	4/5/4/13	45369597	567569	RR	1.24 (1.18-1.31)	0.000	93.4	0.000	0.017
MI	Gaeta et al, 2013	2/3/3/8	41250185	105088	RR	1.25 (1.11-1.39)	0.000	83.2	0.000	0.429
Stroke	Raaby et al, 2017	0/0/5/5	5777211	16110	HR	1.17 (1.07-1.28)	0.000	78.5	0.000	0.343
Hypertension	Duan et al, 2020	10/4/2/16	1069763	126838	OR	1.46 (1.28-1.66)	0.000	94.1	0.000	0.043
Pediatric hypertension	Cho et al, 2021	6/2/0/8	5237538	61384	OR	2.73 (1.79-4.17)	0.000	72.0	0.001	0.638
Pediatric IHD or heart failure	Phan et al, 2020	0/0/3/3	5231916	7145	OR	3.14 (1.08-9.10)	0.035	83.7	0.002	0.687
Atrial fibrillation	Upala et al, 2016	0/0/4/4	4574977	12755	HR	1.42 (1.22-165)	0.000	68.5	0.007	0.255
**Nervous system**										
Dementia	Liu et al, 2020	1/1/5/7	3638487	69414	RR	1.16 (1.06-1.27）	0.001	56.0	0.040	0.685
Non-vascular dementia	Liu et al, 2020	0/0/3/3	3318622	63464	RR	1.13 (1.11-1.16)	0.000	0.0	0.789	0.468
Vascular dementia	Liu et al, 2020	0/0/2/2	103060	601	RR	1.41 (1.09-1.82)	0.009	0.0	0.538	NA
Parkinson's Disease	Ungprasert et al, 2016	1/0/3/4	1130993	46827	OR	1.38 (1.15-1.53)	0.001	35.0	0.200	0.72
**Gastrointestinal system**										
Crohn's disease	Fu et al, 2018	5/0/4/9	7794087	＞19322	OR	1.93 (1.52-2.47)	0.000	85.9	0.000	0.977
Ulcerative colitis	Fu et al, 2018	4/0/4/8	7535087	＞26985	RR	1.77 (1.66-1.88）	0.000	24.3	0.235	0.614
Non-alcoholic fatty liver disease	Candia et al, 2014	6/0/0/6	267761	1994	OR	2.15 (1.57-2.94)	0.000	74.0	0.002	0.710
Helicobacter pylori Infection	Yu et al, 2019	4/4/3/11	2341	787	OR	1.70 (1.15-2.52)	0.008	60.0	0.006	0.154
Hepatitis C	Zhang et al, 2019	2/4/2/8	2051417	3577	OR	1.70 (1.12-2.57)	0.012	84.2	0.000	0.632

Supplementary Table 1. (continued)

Health outcomes	Author, year	Case-control/cross-sectional/cohort/Total studies (n)	Participants (n)	Events (n)	Type of metric	Effect size		Heterogeneity		Small-study effect
						95% CI	*P* Value	I^2	*P* Value	
Celiac disease	Acharya et al, 2019	4/1/2/7	5853094	136536	OR	2.10 (1.66-2.66)	0.000	52.3	0.026	0.202
**Respiratory system**										
COPD	Ungprasert et al, 2015	5/2/0/7	422947	＞19098	OR	1.53 (1.29-1.83)	0.000	81.1	0.000	0.352
Obstructive Sleep Apnea	Ger et al, 2020	2/1/0/3	293564	834	OR	2.60 (1.07-6.32)	0.040	89.3	＜0.0001	0.193
Asthma	Wang et al, 2018	3/0/3/6	644187	35933	OR	1.33 (1.21-1.46)	0.000	52.5	0.061	0.739
**Metabolic diseases**										
Obesity	Armstrong et al, 2012	11/5/0/16	2365369	230248	OR	1.66 (1.46-1.89)	0.000	97.3	0.000	0.653
Diabetes	Mamizadeh et al, 2019	15/11/12/38	13730940	464990	OR	1.69 (1.51-1.89)	＜0.001	98.5	＜0.001	0.087
Metabolic syndrome	Qiao et al, 2021	14/8/0/22	137053	25917	OR	2.02 (1.67-2.43)	＜0.001	83.6	＜0.001	0.119
Pediatric overweight	Phan et al, 2020	6/1/1/8	2563	398	OR	1.54 (1.21-1.95)	0.000	40.8	0.106	0.628
Pediatric hyperlipidemia	Phan et al, 2020	1/0/5/6	5292400	14594	OR	1.84 (1.65-2.04）	0.000	28.8	0.219	0.060
Pediatric metabolic syndrome	Phan et al, 2020	5/0/0/5	61666	310	OR	2.35 (1.75-3.16)	0.000	38.2	0.167	0.044
Pediatric obesity	Cho et al, 2021	11/2/0/13	5239447	77086	OR	2.40 (1.60-3.59)	0.000	91.1	0.001	0.808
Pediatric diabetes	Cho et al, 2021	6/2/0/8	5257716	25656	OR	2.01 (1.09-3.73)	0.026	89.7	0.000	0.985
Pediatric dyslipidemia	Cho et al, 2021	5/2/0/7	5237434	12592	OR	1.67 (1.42-1.97)	0.000	0.0	0.573	0.114
**Pregnancy outcomes**										
Caesarean delivery	Xie et al, 2021	2/0/8/10	6323672	＞1148489	OR	1.35 (1.14-1.60)	0.000	87.1	0.000	0.105
Preterm birth	Xie et al, 2021	0/0/10/10	8614144	＞475541	OR	1.45 (1.21-1.74)	0.000	76.6	0.000	0.007
(Pre)eclampsia	Xie et al, 2021	0/0/8/8	7989798	＞245519	OR	1.28 (1.10-1.49)	0.001	54.7	0.031	0.202
Gestational diabetes	Xie et al, 2021	0/0/5/5	6308489	＞276483	OR	1.21 (1.12-1.31)	0.000	0.0	0.687	0.349
Gestational hypertension	Xie et al, 2021	0/0/4/4	4970699	＞2261	OR	1.28 (1.17-1.40)	0.000	2.7	0.379	0.989
Premature rupture of membranes	Xie et al, 2021	0/0/3/3	1709374	＞693	OR	1.17 (1.06-1.29)	0.002	64.9	0.058	0.373
Prematurity	Xie et al, 2021	0/0/2/2	2350644	＞4	OR	1.12 (1.01-1.25)	0.033	0.0	0.401	NA

Supplementary Table 1. (continued)

Health outcomes	Author, year	Case-control/cross-sectional/cohort/Total studies (n)	Participants (n)	Events (n)	Type of metric	Effect size		Heterogeneity		Small-study effect
						95% CI	*P* Value	I^2	*P* Value	
Congenital malformations	Xie et al, 2021	0/0/4/4	963638	>40432	OR	1.02 (0.91-1.14)	0.793	0.0	0.825	0.207
Neonatal mortality	Xie et al, 2021	0/0/3/3	1756280	>1155	OR	1.14 (0.90-1.43)	0.284	0.0	0.648	0.518
Still birth	Xie et al, 2021	0/0/5/5	3510668	>3630	OR	1.23 (0.93-1.47)	0.518	35.8	0.183	0.665
Spontaneous abortion	Xie et al, 2021	0/0/4/4	48576	>288	OR	1.44 (0.95-2.17)	0.086	77.3	0.004	0.274
Ante- or postpartum hemorrhage	Xie et al, 2021	0/0/3/3	3302340	>2392	OR	1.12 (0.71-1.76)	0.630	91.2	0.000	0.785
Low birth weight	Xie et al, 2021	0/0/5/5	2365757	>841	OR	1.15 (0.93-1.42)	0.456	70.0	0.471	0.94
Small for gestational age	Xie et al, 2021	0/0/7/7	704746	>257327	OR	1.05 (0.99-1.12)	0.129	15.6	0.314	0.725
**Other outcomes**										
CKD	Ungprasert et al, 2018	0/0/4/4	919436	199808	RR	1.34 (1.14-1.58)	0.000	95.8	0.000	0.044
End-stage CKD	Ungprasert et al, 2018	0/0/3/3	864949	151729	RR	1.29 (1.05-1.60)	0.015	12.6	0.318	0.427
Uveitis	Li et al, 2020	0/0/4/4	263187128	>15004	RR	1.50 (1.08-2.08)	0.015	91.0	0.000	0.466
Fracture	Chen et al, 2020	0/0/5/5	1006349	67233	OR	1.09 (1.06-1.11)	0.000	0.0	0.434	0.674
Geographic tongue	Alvarez et al, 2019	11/0/0/11	12327	784	OR	3.31 (2.66-4.11）	0.000	48.2	0.037	0.198
Multiple sclerosis	Islam et al, 2019	4/0/5/9	NR	53863	RR	1.67 (1.46-1.91)	<0.0001	0.0	0.486	0.443
Erectile dysfunction	Wu et al, 2018	2/6/1/9	1693957	87545	OR	1.93 (1.51-2.46)	<0.0001	86.0	<0.0001	0.036
Aortic aneurysm	Yu et al, 2020	0/0/3/3	5731399	24864	HR	1.30 (1.10-1.55）	0.002	53.5	0.117	0.426
Schizophrenia	Ungprasert et al, 2018	4/0/1/5	9271621	405421	OR	1.35 (1.20-1.53)	0.000	33.1	0.201	0.345
Prevalence of depression	Zusman et al, 2020	0/1/3/4	23794	>178	OR	1.69 (1.37-2.08)	0.000	96.7	0.000	0.911
Incidence of depression	Zusman et al, 2020	0/0/4/4	28614	>5270	RR	1.44 (1.20-1.73)	0.000	94.7	0.000	0.759
Prevalence of anxiety	Zusman et al, 2020	0/0/2/2	16128	>122	OR	1.48 (1.34-1.64)	0.000	0.0	0.780	NA
Osteoporosis	Sepehri et al, 2021	3/2/2/7	198774970	1672184	OR	1.28 (0.86-1.90)	0.225	99.2	0.000	0.483
Osteopenia	Sepehri et al, 2021	0/2/0/2	198535032	309110	OR	1.50 (0.74-3.02)	0.260	99.5	0.000	0.601
Suicide	Chi et al, 2017	0/0/2/2	527967	1074	RR	1.13 (0.87-1.46)	0.360	57.0	0.130	NA

Supplementary Table 1. (continued)

Health outcomes	Author, year	Case-control/cross-sectional/cohort/Total studies (n)	Participants (n)	Events(n)	Type of metric	Effect size		Heterogeneity		Small-study effect
						95% CI	P Value	I2	P Value	
Suicide attempt	Chi et al, 2017	0/0/3/3	720274	2102	RR	1.25 (0.89-1.75)	0.201	92.7	＜0.0001	0.339
VTE	Hillary et al, 2021	0/0/5/5	48731075	86830	RR	1.29 (0.92-1.80)	0.139	96.8	0.000	0.281

Abbreviation: ACM, all-cause mortality; CVD, cardiovascular disease; MI, myocardial infarction; IHD, ischemic heart disease; COPD, chronic obstructive pulmonary disease; CKD, chronic kidneys disease; VTE, Venous Thromboembolism.
