# Supplementary material for: Psoriasis and medical ramifications: A comprehensive analysis based on observational meta-analyses
Source: Front Med (Lausanne). 2022 Aug 29;9:998815. doi: 10.3389/fmed.2022.998815 (PMC9465012; doi:10.3389/fmed.2022.998815)
Supplement: Supplementary file 2 [file Table_2.pdf]

Supplementary Table 2. The strength of epidemiologic evidence of 85 unique health outcomes.

Health outcomes	Author, year	Precision of the estimate		Consistency of results	No evidence of small-study effects ($P > 0.1$)	Grade
		>1000 disease cases	$P<0.001$	$I^2 < 50\%$ and Cochran Q test $P > .10$		
**Mortality**						
ACM	Dhana et al，2019	Yes	Yes	No	Yes	Moderate
CVD mortality	Dhana et al，2019	Yes	Yes	No	Yes	Moderate
Mortality in liver disease	Dhana et al，2019	No	Yes	No	No	Weak
Mortality in respiratory disease	Dhana et al，2019	No	No	Yes	No	Weak
Mortality in kidney disease	Dhana et al，2019	No	Yes	No	No	Weak
Mortality in infections	Dhana et al，2019	No	Yes	Yes	No	Weak
Mortality in malignancy	Dhana et al，2019	Yes	No (P＞0.05)	Yes	No	NA
Mortality in cancer	Trafford et al, 2019	Yes	No (P＞0.05)	Yes	No	NA
**Cancer**						
Cancer incidence	Trafford et al, 2019	Yes	No	No	Yes	Weak
Respiratory tract cancer	Pouplard et al, 2013	No	No	No	No	Weak
Upper aerodigestive tract	Pouplard et al, 2013	No	Yes	Yes	Yes	Moderate
Urinary tract cancer	Pouplard et al, 2013	No	No	Yes	Yes	Weak
Colorectal cancer	Fu et al, 2020	Yes	Yes	Yes	No	Moderate
Colon cancer	Fu et al, 2020	Yes	No	Yes	No	Weak
Hematologic malignancy	Bellinato et al, 2021	No	Yes	Yes	Yes	Moderate
Lymphoma	Bellinato et al, 2021	No	No	No	Yes	Weak
Hodgkin lymphoma	Bellinato et al, 2021	No	Yes	Yes	Yes	Moderate
Non-Hodgkin lymphoma	Bellinato et al, 2021	No	No	No	Yes	Weak
Cutaneous T-cell lymphoma	Bellinato et al, 2021	No	Yes	No	Yes	Weak
Multiple myeloma	Bellinato et al, 2021	No	No	Yes	No	Weak
Leukemia	Bellinato et al, 2021	Yes	No	No	Yes	Weak

Supplementary Table 2. (continued)

Health outcomes	Author, year	Precision of the estimate		Consistency of results	No evidence of small-study effects ($P > 0.1$)	Grade
		>1000 disease cases	P<0.001	$I^2 < 50\%$ and Cochran Q test $P > .10$		
Non-melanoma skin cancer	Wang et al, 2020	Yes	Yes	No	Yes	Moderate
Squamous cell carcinoma	Pouplard et al, 2013	No	Yes	No	Yes	Weak
Rectal cancer	Fu et al, 2020	No	No (P>0.05)	Yes	Yes	NA
Melanoma	Pouplard et al, 2013	No	No (P>0.05)	Yes	Yes	NA
**Cardiovascular system**						
CVD	Gaeta et al, 2013	Yes	Yes	No	No	Weak
MI	Gaeta et al, 2013	Yes	Yes	No	Yes	Moderate
Stroke	Raaby et al, 2017	Yes	Yes	No	Yes	Moderate
Hypertension	Duan et al, 2020	Yes	Yes	No	No	Weak
Pediatric hypertension	Cho et al	Yes	Yes	No	Yes	Moderate
Pediatric IHD or heart failure	Phan et al, 2020	Yes	No	No	Yes	Weak
Atrial fibrillation	Upala et al, 2016	Yes	Yes	No	Yes	Moderate
**Nervous system**						
Dementia	Liu et al, 2020	Yes	No	No	Yes	Weak
Non-vascular dementia	Liu et al, 2020	Yes	Yes	Yes	Yes	High
Vascular dementia	Liu et al, 2020	No	No	Yes	No	Weak
Parkinson's Disease	Ungprasert et al, 2016	Yes	No	Yes	Yes	Weak
**Gastrointestinal system**						
Crohn's disease	Fu et al, 2018	Yes	Yes	No	Yes	Moderate
Ulcerative colitis	Fu et al, 2018	Yes	Yes	Yes	Yes	High
Non-alcoholic fatty liver disease	Candia et al, 2014	Yes	Yes	No	Yes	Moderate
Helicobacter pylori Infection	Yu et al, 2019	No	No	No	Yes	Weak
Hepatitis C	Zhang et al. 2019	Yes	No	No	Yes	Weak

Supplementary Table 2. (continued)

Health outcomes	Author, year	Precision of the estimate		Consistency of results	No evidence of small-study effects ($P > 0.1$)	Grade
		>1000 disease cases	$P<0.001$	$I^2 < 50\%$ and Cochran Q test $P > .10$		
Celiac	Acharya et al, 2019	Yes	Yes	No	Yes	Moderate
**Respiratory system**						
COPD	Ungprasert et al, 2015	Yes	Yes	No	Yes	Moderate
Obstructive Sleep Apnea	Ger et al, 2020	No	No	No	Yes	Weak
Asthma	Wang et al, 2018	Yes	Yes	No	Yes	Moderate
**Metabolic diseases**						
Obesity	Armstrong et al, 2012	Yes	Yes	No	Yes	Moderate
Diabetes	Mamizadeh et al, 2019	Yes	Yes	No	No	Weak
Metabolic syndrome	Qiao et al, 2021	Yes	Yes	No	Yes	Moderate
Pediatric overweight	Phan et al, 2020	No	Yes	Yes	Yes	Moderate
Pediatric hyperlipidemia	Phan et al, 2020	Yes	Yes	Yes	No	Moderate
Pediatric metabolic syndrome	Phan et al, 2020	No	Yes	Yes	No	Weak
Pediatric obesity	Cho et al, 2021	Yes	Yes	No	Yes	Moderate
Pediatric diabetes	Cho et al, 2021	Yes	No	No	Yes	Weak
Pediatric dyslipidemia	Cho et al, 2021	Yes	Yes	Yes	Yes	High
**Pregnancy outcomes**						
Caesarean delivery	Xie et al, 2021	Yes	Yes	No	Yes	Moderate
Preterm birth	Xie et al, 2021	Yes	Yes	No	No	Weak
(Pre)eclampsia	Xie et al, 2021	Yes	No	No	Yes	Weak
Gestational diabetes	Xie et al, 2021	Yes	Yes	Yes	Yes	High
Gestational hypertension	Xie et al, 2021	Yes	Yes	Yes	Yes	High
Premature rupture of membranes	Xie et al, 2021	No	No	No	Yes	Weak
Prematurity	Xie et al, 2021	No	No	Yes	No	Weak

Supplementary Table 2. (continued)

Health outcomes	Author, year	Precision of the estimate		Consistency of results	No evidence of small-study effects ($P > 0.1$)	Grade
		>1000 disease cases	$P<0.001$	$I^2 < 50\%$ and Cochran Q test $P > .10$		
Congenital malformations	Xie et al, 2021	Yes	No ($P>0.05$)	Yes	Yes	NA
Neonatal mortality	Xie et al, 2021	Yes	No ($P>0.05$)	Yes	Yes	NA
Still birth	Xie et al, 2021	Yes	No ($P>0.05$)	Yes	Yes	NA
Spontaneous abortion	Xie et al, 2021	No	No ($P>0.05$)	No	Yes	NA
Ante- or postpartum hemorrhage	Xie et al, 2021	Yes	No ($P>0.05$)	No	Yes	NA
Low birth weight	Xie et al, 2021	No	No ($P>0.05$)	No	Yes	NA
Small for gestational age	Xie et al, 2021	Yes	No ($P>0.05$)	Yes	Yes	NA
**Other outcomes**						
CKD	Ungprasert et al, 2018	Yes	Yes	No	No	Weak
End-stage CKD	Ungprasert et al, 2018	Yes	No	Yes	Yes	Weak
Uveitis	Li et al, 2020	Yes	No	No	Yes	Weak
Fracture	Chen et al, 2020	Yes	Yes	Yes	Yes	High
Geographic tongue	Alvarez et al, 2019	No	Yes	No	Yes	Weak
Multiple sclerosis	Islam et al, 2019	Yes	Yes	Yes	Yes	High
Erectile dysfunction	Wuet et al, 2018	Yes	Yes	No	No	Weak
Aortic aneurysm	Yu et al, 2020	Yes	No	No	Yes	Weak
Schizophrenia	Ungprasert et al, 2018	Yes	Yes	Yes	Yes	High
Prevalence of depression	Zusman et al, 2020	No	Yes	No	Yes	Weak
Incidence of depression	Zusman et al, 2020	Yes	Yes	No	Yes	Moderate
Prevalence of anxiety	Zusman et al, 2020	No	Yes	Yes	No	Weak
Osteoporosis	Sepehri et al, 2021	Yes	No ($P>0.05$)	No	Yes	NA
Osteopenia	Sepehri et al, 2021	Yes	No ($P>0.05$)	No	Yes	NA
Suicide	Chi et al, 2017	Yes	No ($P>0.05$)	No	No	NA

Supplementary Table 2. (continued)

Health outcomes	Author, year	Precision of the estimate		Consistency of results	No evidence of small-study effects ($P > 0.1$)	Grade
		>1000 disease cases	$P<0.001$	$I^2 < 50\%$ and Cochran Q test $P > .10$		
Suicide attempt	Chi et al, 2017	Yes	No ($P>0.05$)	No	Yes	NA
VTE	Hillary et al, 2021	Yes	No ($P>0.05$)	No	Yes	NA

Abbreviations: ACM, all-cause mortality; CVD, cardiovascular disease; MI, myocardial infarction; IHD, ischemic heart disease; COPD, chronic obstructive pulmonary disease; CKD, chronic kidneys disease; VTE, Venous Thromboembolism.

NOTE. The strength of epidemiologic evidence was rated as follows:

High, if all criteria were satisfied: precision of the estimate (P < .001 and >1000 disease cases), consistency of results (I2 < 50% and Cochran Q test P > .10), and no evidence of small-study effects (P > .10).

Moderate, if a maximum of 1 criterion was not satisfied and a P < .001 was found.

Weak, in other cases (P < .05).

NA, P values are greater than 0.05, so the epidemiologic quality of these meta cannot be rated.
