# Supplementary material for: Psoriasis and medical ramifications: A comprehensive analysis based on observational meta-analyses
Source: Front Med (Lausanne). 2022 Aug 29;9:998815. doi: 10.3389/fmed.2022.998815 (PMC9465012; doi:10.3389/fmed.2022.998815)
Supplement: Supplementary file 3 [file Table_3.pdf]

Supplementary Table 3. Assessments of AMSTAR2 scores.

Reference	AMSTAR 2 checklist																Overall assessment quality
	NO.1	NO.2	NO.3	NO.4	NO.5	NO.6	NO.7	NO.8	NO.9	NO.10	NO.11	NO.12	NO.13	NO.14	NO.15	NO.16	
DhanTa et al，2019	Y	N	Y	pY	Y	Y	N	Y	Y	N	Y	Y	Y	Y	Y	Y	Critically low
Trafford et al, 2019	Y	N	Y	pY	N	N	N	Y	Y	N	N	N	N	Y	N	Y	Critically low
Pouplard et al, 2013	Y	N	Y	pY	Y	Y	N	Y	Y	N	Y	N	Y	Y	Y	Y	Critically low
Fu et al, 2020	Y	Y	Y	Y	Y	Y	N	Y	Y	N	Y	N	N	N	N	Y	Critically low
Bellinato et al, 2021	Y	Y	Y	pY	Y	Y	N	Y	Y	N	N	Y	Y	Y	Y	Y	Critically low
Wang et al, 2020	Y	N	Y	Y	Y	Y	N	Y	Y	N	Y	Y	Y	Y	Y	Y	Critically low
Gaeta et al, 2013	Y	N	Y	pY	Y	Y	N	Y	Y	N	Y	Y	N	N	Y	Y	Critically low
Raaby et al, 2017	N	N	Y	pY	Y	Y	N	Y	Y	N	N	N	N	N	N	Y	Critically low
Duan et al, 2020	Y	N	Y	Y	Y	Y	N	Y	Y	N	Y	Y	Y	Y	Y	Y	Critically low
Cho et al, 2021	Y	N	Y	pY	Y	Y	N	Y	Y	N	N	N	Y	Y	Y	Y	Critically low
Phan et al, 2020	Y	N	Y	Y	N	N	N	Y	N	N	N	N	Y	Y	N	N	Critically low
Upala et al, 2016	Y	N	Y	pY	Y	Y	N	Y	Y	N	Y	Y	Y	N	Y	Y	Critically low
Liu et al, 2020	Y	N	Y	Y	Y	Y	N	Y	Y	N	Y	Y	Y	N	Y	Y	Critically low
Ungprasert et al, 2016	Y	N	Y	pY	Y	Y	N	Y	Y	N	Y	Y	Y	N	Y	Y	Critically low
Fu et al, 2018	Y	N	Y	pY	Y	Y	N	Y	Y	N	Y	N	N	N	N	Y	Critically low
Candia et al, 2014	Y	N	Y	Y	Y	Y	N	Y	Y	N	Y	N	Y	Y	Y	Y	Critically low
Yu et al, 2019	Y	N	Y	pY	Y	Y	N	N	Y	N	Y	Y	Y	Y	Y	Y	Critically low
Zhang et al. 2019	Y	N	Y	pY	N	N	N	N	Y	N	Y	N	Y	N	Y	N	Critically low
Acharya et al, 2019	N	N	Y	pY	Y	Y	N	Y	Y	N	Y	Y	N	Y	Y	Y	Critically low
Ungprasert et al, 2015	Y	N	Y	pY	Y	Y	N	Y	Y	N	Y	Y	Y	Y	Y	Y	Critically low
Ger et al, 2020	Y	Y	Y	Y	Y	Y	N	Y	Y	N	Y	Y	Y	Y	Y	Y	Low
Wang et al, 2018	Y	N	Y	pY	Y	Y	N	N	Y	N	Y	Y	Y	Y	Y	Y	Critically low

Supplementary table 3. (continued)

Reference	AMSTAR 2 checklist																Overall assessment quality
	NO.1	NO.2	NO.3	NO.4	NO.5	NO.6	NO.7	NO.8	NO.9	NO.10	NO.11	NO.12	NO.13	NO.14	NO.15	NO.16	
Armstrong et al, 2012	Y	N	Y	pY	Y	Y	Y	Y	Y	N	Y	N	N	N	Y	Y	Critically low
Mamizadeh et al, 2019	Y	Y	Y	Y	N	N	N	Y	Y	N	Y	Y	Y	Y	Y	Y	Low
Qiao et al, 2021	Y	N	Y	pY	Y	Y	N	N	N	N	Y	N	N	Y	Y	Y	Critically low
Xie et al, 2021	Y	N	Y	Y	Y	Y	N	Y	Y	N	N	Y	Y	Y	Y	Y	Critically low
Ungprasert et al, 2018	Y	N	Y	pY	Y	Y	N	Y	Y	N	Y	Y	Y	Y	Y	Y	Critically low
Li et al, 2020	Y	N	Y	pY	Y	Y	N	Y	Y	N	Y	Y	Y	Y	Y	Y	Critically low
Chen et al, 2020	Y	Y	Y	Y	Y	Y	N	Y	Y	N	Y	Y	Y	Y	Y	Y	Low
Alvarez et al, 2019	Y	N	Y	pY	Y	Y	N	N	Y	N	Y	Y	Y	Y	Y	Y	Critically low
Islam et al, 2019	Y	N	Y	pY	Y	Y	N	N	Y	N	Y	Y	Y	Y	Y	Y	Critically low
Wu et al, 2018	Y	N	Y	pY	Y	Y	N	Y	Y	N	Y	Y	Y	Y	Y	Y	Critically low
Yu et al, 2020	Y	N	Y	pY	Y	N	N	Y	Y	N	Y	N	N	Y	N	Y	Critically low
Ungprasert et al, 2018	Y	N	Y	pY	Y	Y	N	N	Y	N	Y	Y	Y	N	Y	Y	Critically low
Zusman et al, 2020	Y	N	Y	pY	N	N	N	Y	Y	N	Y	N	N	Y	N	Y	Critically low
Sepehri et al, 2021	Y	N	Y	pY	Y	Y	N	N	Y	N	Y	N	Y	N	Y	Y	Critically low
Chi et al, 2017	Y	N	Y	pY	N	N	N	N	Y	N	Y	N	N	N	Y	Y	Critically low
Hillary et al, 2021	Y	Y	Y	Y	Y	N	Y	Y	Y	N	Y	N	Y	N	N	Y	Low

AMSTAR 2 checklist (items in italic are considered critical):

1, PICO description; 2, protocol registered before the commencement of the review; 3, study design included in the review; 4, adequacy of the literature search; 5, two authors study selection; 6, two authors study extraction; 7, list for excluding individual studies; 8, included studies descripted in detail; 9, risk of bias for the single studies that included in the review; 10, source of funding of primary studies; 11, appropriateness of meta-analytical methods; 12, impact of risk of bias of single studies on the results of the meta-analysis; 13, consideration of risk of bias when interpreting the results of the review; 14 explanation and discussion of the heterogeneity observed; 15, assessment of presence and likely impact of publication bias; 16, funding sources and conflict of interest declared.

Abbreviations: Y, yes; pY, partial yes; N, no.

Footnotes:

High: 0–1 non-critical weakness. The systematic review provides an accurate and comprehensive summary of the results of the available studies that address the question of interest.

Moderate: >1 non-critical weakness. The systematic review has more than one weakness, but no critical flaws. It may provide an accurate summary of the results of the available studies that were included in the review.

Low: 1 critical flaw with or without non-critical weaknesses. The review has a critical flaw and may not provide an accurate and comprehensive summary of the available studies that address the question of interest.

Critically low: >1 critical flaw with or without non-critical weaknesses. The review has more than one critical flaw and should not be relied on to provide an accurate and comprehensive summary of the available studies.

No2, 4, 7, 9, 11, 13, 15 are the critical items.
